# The relationship between cerebral regional oxygen saturation during extracorporeal cardiopulmonary resuscitation and the neurological outcome in a retrospective analysis of 16 cases

**DOI:** 10.1186/s40560-017-0216-1

**Published:** 2017-02-23

**Authors:** Naoki Ehara, Tomoya Hirose, Tadahiko Shiozaki, Akinori Wakai, Tetsuro Nishimura, Nobuto Mori, Mitsuo Ohnishi, Daikai Sadamitsu, Takeshi Shimazu

**Affiliations:** 1 Traumatology and Critical Care Medical Center, National Hospital Organization Osaka National Hospital, 2-1-14 Hoenzaka Chuo-ku, Osaka, Osaka 540-0006 Japan; 2 Department of Traumatology and Acute Critical Medicine, Osaka University Graduate School of Medicine, 2-15 Yamadaoka, Suita, Osaka 565-0871 Japan

**Keywords:** Cerebral regional oxygen saturation, Extracorporeal cardiopulmonary resuscitation, Neurological outcome, Out-of-hospital cardiac arrest, Near-infrared spectroscopy

## Abstract

**Background:**

In recent years, the measurement of cerebral regional oxygen saturation (rSO_2_) during resuscitation has attracted attention. The objective of this study was to clarify the relationship between the serial changes in the cerebral rSO_2_ values during extracorporeal cardiopulmonary resuscitation (ECPR) and the neurological outcome.

**Methods:**

We measured the serial changes in the cerebral rSO_2_ values of patients with out-of-hospital cardiac arrest before and after ECPR in Osaka National Hospital.

**Results:**

From January 2013 through March 2015, the serial changes in the cerebral rSO_2_ values were evaluated in 16 patients. Their outcomes, as measured by the Glasgow Outcome Scale (GOS) score at discharge, included good recovery (GR) (*n* = 4), vegetative state (VS) (*n* = 2), and death (D) (*n* = 10). In the poor neurological group (VS and D: *n* = 12; age, 52.8 ± 4.0 years), the cerebral rSO_2_ values showed a significant increase during ECPR (5 min before ECPR: 52.0 ± 1.8%; 2 min before ECPR: 56.1 ± 2.3%; 2 min after ECPR: 63.5 ± 2.2%; 5 min after ECPR: 66.4 ± 2.2%; 10 min after ECPR: 67.6 ± 2.3% [*P* < 0.01]). In contrast, in the good neurological group (GR: *n* = 4; age, 53.8 ± 6.9 years), the cerebral rSO_2_ values did not increase to a significant extent during ECPR (5 min before ECPR: 61.9 ± 3.1%; 2 min before ECPR: 57.1 ± 4.0%; 2 min after ECPR: 59.6 ± 3.8%; 5 min after ECPR: 61.0 ± 3.7%; 10 min after ECPR: 62.0 ± 3.8% [*P* = 0.88]). Our study suggested that the patients whose cerebral rSO_2_ values showed no significant improvement after ECPR might have had a good neurological prognosis.

**Conclusions:**

The serial changes in the cerebral rSO_2_ values during ECPR may predict a patient’s neurological outcome. The further evaluation of the validity of rSO_2_ monitoring during ECPR may lead to a new resuscitation strategy.

## Background

Sudden cardiac arrest is one of the most important causes of death and an important public health problem in the industrialised world [1]. However, survival from out-of-hospital cardiac arrest (OHCA) is still low [2], and to improve it, we think that a new resuscitation strategy is needed.

In recent years, the measurement of cerebral regional oxygen saturation (rSO_2_) by near-infrared spectroscopy (NIRS) during resuscitation has attracted attention. We have already reported the serial changes in the cerebral rSO_2_ values during resuscitation in patients with OHCA [3]. Chest compression alone could not increase the cerebral rSO_2_ value, which was found to gradually increase with a return of spontaneous circulation (ROSC). The cerebral rSO_2_ value also increased promptly after the initiation of extracorporeal cardiopulmonary resuscitation (ECPR) [4]. However, we could not predict the neurological outcome by evaluating the cerebral rSO_2_ value in patients with OHCA in 2010 [4].

It is challenging to predict the neurological outcome following OHCA. Although some researchers have reported that the cerebral rSO_2_ value at hospital arrival can predict the neurological outcome in patients with OHCA [5–7], we thought that this conclusion was incorrect. Because the rSO_2_ values always change depending on the patient’s situation at the time when the cerebral rSO_2_ is measured [4, 8], we hypothesise that the serial change in the rSO_2_ values is important rather than a single measured value.

By chance, we detected a difference in the way the values of cerebral rSO_2_ in patients with OHCA changed before and after the application of ECPR. Thus, the objective of this study was to clarify the relationship between the serial changes in the cerebral rSO_2_ value during ECPR and the neurological outcome.

## Methods

### Study design and data collection

This retrospective study was approved by the Ethics Committee of National Hospital Organization Osaka National Hospital (Osaka, Japan). The subjects were all cardiopulmonary arrest (CPA) patients who were transferred to National Hospital Organization Osaka National Hospital by emergency life-saving technicians (ELTs). At the emergency department, a sensor was attached to the patient’s forehead to continuously monitor the cerebral rSO_2_ value during resuscitation. ELTs and medical staff performed cardiopulmonary resuscitation according to the recommendations of the Japan Resuscitation Council Guidelines 2010, which were based on the American Heart Association and the International Liaison Committee on Resuscitation guidelines [9]. The medical staff could see the rSO_2_ values during resuscitation, and the values were automatically recorded. However, they did not change the treatment according to the cerebral rSO_2_ data. We retrospectively collected and analysed data from the CPA patients undergoing ECPR.

The variables that were analysed included the age, sex, initial rhythm, whether the OHCA was witnessed, and whether a bystander performed cardiopulmonary resuscitation (CPR). We evaluated the patient’s cerebral rSO_2_ values at 5 and 2 min before the application of ECPR and at 2, 5, and 10 min after the application of ECPR. The neurological prognosis was evaluated according to the Glasgow Outcome Scale (GOS) score. The normal range of cerebral rSO_2_ was determined from 15 healthy adult volunteers whose values were measured on room air.

### The NIRS-based rSO_2_ monitoring system

An rSO_2_ monitor (TOS-OR; TOSTEC Co., Tokyo, Japan) was used to measure the cerebral rSO_2_ value. The monitor measures the oxygen saturation based on the Beer-Lambert law, using three different wavelengths of near-infrared LED light, which have specific absorbance in oxyhaemoglobin and deoxyhaemoglobin. The lights pass through the skin to a depth of approximately 3 cm, and the reflected lights are sensed by a photodiode. The reflected lights mainly represent the haemoglobin information in the cerebral cortex. The system can measure rSO_2_ data every second without the need for pulsation. It is therefore possible to continuously monitor the rSO_2_ values of CPA patients.

### Data analysis

Two rSO_2_ values (on the left side and right side) were acquired continuously. The average of these two values was then calculated. If the value of one of the two values appeared to be in error, then the other value was used for the data analysis. Finally, graphs were drawn of the serial changes in the cerebral rSO_2_ values.

### Statistical analysis

All of the data are represented as the mean ± standard deviation (SD). The Wilcoxon rank sum test was used to compare the differences between the two groups at each measurement point. A one-way repeated-measures analysis of variance (ANOVA) was used to evaluate the differences among the measured points. *P* values of <0.05 were considered to indicate statistical significance. All of the statistical analyses were performed using the JMP Pro 11 for Windows software program (SAS Institute Inc., Cary, NC, USA).

## Results

### Normal range of cerebral rSO_2_

The normal range of cerebral rSO_2_ in the healthy adult volunteers (*n* = 15; age, 43.2 ± 8.9 years; 10 men and 5 women) was 71.2 ± 3.9% (on room air).

### Patient characteristics

From January 2013 through March 2015, the serial changes in the cerebral rSO_2_ values of 16 patients were evaluated. Their outcomes, as measured by the GOS score at discharge, included a good recovery (GR) (*n* = 4), vegetative state (VS) (*n* = 2), and death (D) (*n* = 10). The time from the onset of cardiac arrest to the initiation of ECPR did not differ to a statistically significant extent between the poor neurological group (VS and D: 64.6 ± 22.6 min) and the good neurological group (GR: 49.5 ± 5.7 min) (*P* = 0.11). The characteristics of the patients with OHCA are shown in Table 1.

**Table 1:** The characteristics of the patients with out-of-hospital cardiac arrest

	Good neurological outcome group (GR)	Poor neurological outcome group (VS and D)
Number	4	12
Age (±SD) (years)	53.8 ± 6.9	52.9 ± 4.0
Male (%)	4 (100%)	10 (83.3%)
Initial rhythm		
VF (%)	4 (100%)	6 (50.0%)
PEA (%)	0	4 (33.3%)
Asystole (%)	0	2 (16.7%)
Witnessed		
Yes (%)	4 (100%)	11 (91.7%)
No (%)	0	1 (8.3%)
Bystander CPR		
Yes (%)	4 (100%)	10 (83.3%)
No (%)	0	2 (16.7%)
The time from the onset of cardiac arrest to the initiation of ECPR (min)	49.5 ± 5.7	64.6 ± 22.6

*CPR* cardiopulmonary resuscitation, *D* death, *GR* good recovery, *PEA* pulseless electrical activity, *VF* ventricular fibrillation, *VS* vegetative state

### The relationship between the cerebral rSO_2_ values during ECPR and the neurological outcome

**Fig. 1:**
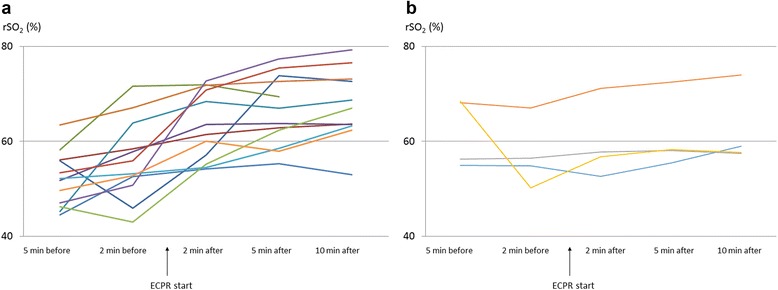
The serial change in the cerebral regional oxygen saturation (rSO_2_) of each patient during extracorporeal cardiopulmonary resuscitation (ECPR). **a** The poor neurological outcome group (*n* = 12) and **b** the good neurological outcome group (*n* = 4)

**Fig. 2:**
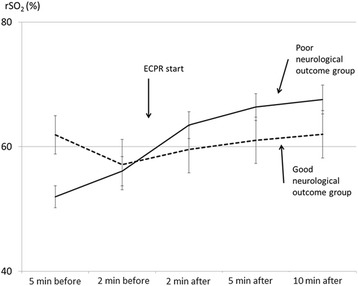
The relationship between cerebral regional oxygen saturation (rSO_2_) during extracorporeal cardiopulmonary resuscitation (ECPR) and the neurological outcome

The serial changes in the cerebral rSO_2_ values during ECPR for each patient are shown in Fig. 1. The serial changes in the mean cerebral rSO_2_ value during ECPR in the good and poor neurological outcome groups are shown in Fig. 2. The only significant difference in the cerebral rSO_2_ values of the two groups was observed at 5 min before ECPR (*P* < 0.05) (2 min before ECPR: *P* = 0.95; 2 min after ECPR: *P* = 0.36; 5 min after ECPR: *P* = 0.20; and 10 min after ECPR: *P* = 0.21). In the poor neurological group (VS and D: *n* = 12; age, 52.8 ± 4.0 years), the cerebral rSO_2_ values increased significantly during ECPR (5 min before ECPR: 52.0 ± 1.8%; 2 min before ECPR: 56.1 ± 2.3%; 2 min after ECPR: 63.5 ± 2.2%; 5 min after ECPR: 66.4 ± 2.2%; and 10 min after ECPR: 67.6 ± 2.3% [*P* < 0.01]) (Figs. 1a and 2). In contrast, in the good neurological group (GR: *n* = 4; age, 53.8 ± 6.9 years), the cerebral rSO_2_ values did not increase to a statistically significant extent during ECPR (5 min before ECPR: 61.9 ± 3.1%; 2 min before ECPR: 57.1 ± 4.0%; 2 min after ECPR: 59.6 ± 3.8%; 5 min after ECPR: 61.0 ± 3.7%; and 10 min after ECPR: 62.0 ± 3.8% [*P* = 0.88]) (Figs. 1b and 2).

## Discussion

Recently, a systematic review and meta-analysis reported by Sanfilippo et al. [10] showed that higher initial and average cerebral rSO_2_ values were both associated with a greater chance of achieving an ROSC in patients with cardiac arrest; however, they could not show a relationship between the cerebral rSO_2_ value and the neurological outcome of patients resuscitated from cardiac arrest. Both Ito et al. [5] and Storm et al. [11] revealed significantly higher cerebral rSO_2_ values on hospital arrival in patients with a good neurological outcome, but the cerebral rSO_2_ values of the good and poor outcome groups varied widely and there was a large amount of overlap. We hypothesised that a single measurement of cerebral value might not be important because the rSO_2_ values always change depending on the patient’s situation at the time of the rSO_2_ measurement [4, 8]. Therefore, we think that the value of NIRS should be assessed by trend value, not by absolute value.

Counterintuitively, the results of this study suggested that the patients whose cerebral rSO_2_ values did not show a significant improvement after ECPR might have had a good neurological prognosis (Figs. 1b and 2). We thought that, in the good neurological group, the brain blood flow was recovered by ECPR, the oxygen was delivered to brain tissue, and the brain tissue might start to consume oxygen. These events continuously change. So, we believe that the most important factor when evaluating the cerebral rSO_2_ value during resuscitation to predict the neurological outcome is serial change in the cerebral rSO_2_ values. Two reports have shown a significant increase in the cerebral rSO_2_ value after the application of ECPR in patients with OHCA; however, all of the reported patients had a poor neurological outcome or died [4, 12]. One report showed that the tissue oxygen index decreased in a patient with a favourable neurological outcome (*n* = 1) but that it did not change in patients with unfavourable neurological outcome (*n* = 14) [13]. These reports also failed to show a relationship between the cerebral rSO_2_ value during ECPR and the neurological outcome.

**Fig. 3:**
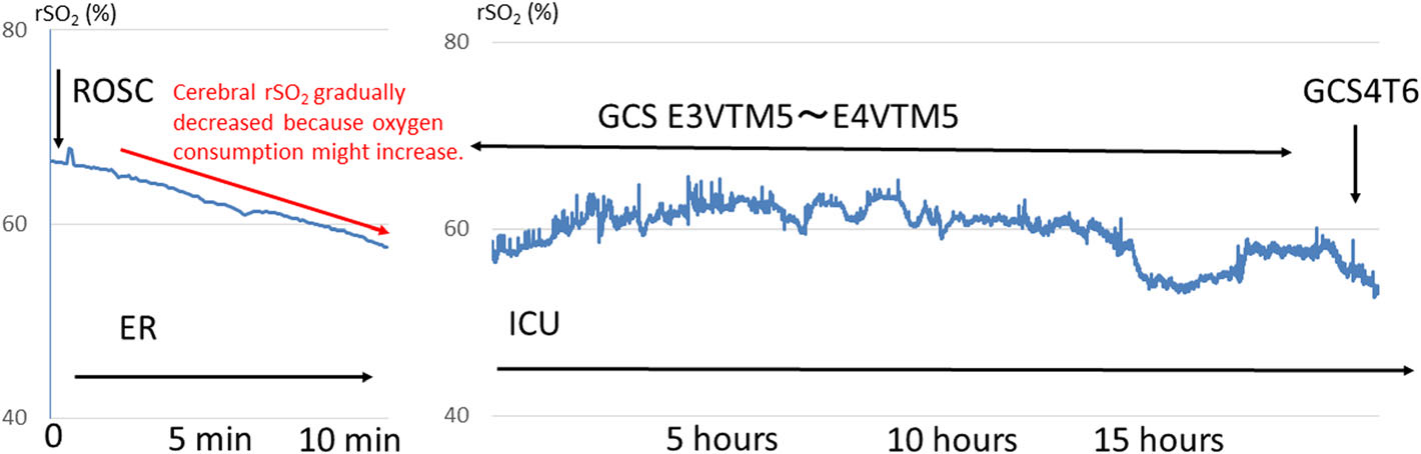
The serial change in the cerebral regional oxygen saturation of a 74-year-old male patient during ICU treatment after the ROSC. When we started cerebral rSO_2_ measurement in this patient, the cerebral rSO_2_ value was 66%; it gradually fell to 57% after 12 min. His neurological outcome was good. We think that the ROSC led to the recovery of the cerebral blood flow, and because the oxygen consumption of patients with a good neurological outcome might increase, the cerebral rSO_2_ value can be expected to decrease. *ER* emergency room, *GCS* Glasgow Coma Scale, *ICU* intensive care unit, *ROSC* return of spontaneous circulation, *rSO*_*2*_ regional saturation of oxygen

We hypothesised that the cerebral rSO_2_ values of the patients in the good neurological outcome group did not change before or after ECPR because their brain tissue might have been consuming oxygen; therefore, we began to evaluate the cerebral rSO_2_ value during intensive care unit (ICU) treatment after the ROSC. In Fig. 3, we show one patient with an ROSC who displayed a decreasing cerebral rSO_2_ value but who experienced a GR. When we started to perform cerebral rSO_2_ measurement in this patient, his cerebral rSO_2_ value was 66%, and it gradually dropped to 57% after 12 min. His neurological outcome was good. We think that the ROSC led to the recovery of the cerebral blood flow, and because in patients with a good neurological outcome oxygen consumption might increase, as a result, his cerebral rSO_2_ value decreased. Our study investigates the serial changes in the cerebral rSO_2_ value during ICU after an ROSC treatment is currently ongoing. In the future, additional studies should be performed to investigate the relationship between the serial changes in the cerebral rSO_2_ value after an ROSC and the neurological outcome.

A recent review on ECPR revealed that the outcome of ECPR in patients with in-hospital cardiac arrest was satisfactory, with good survival rates and good neurological outcome [14]. However, it is more challenging to achieve satisfactory ECPR results in OHCA patients, and a good outcome can only be obtained in 15–20% of the patients, provided that the time from cardiac arrest to the initiation of ECPR is shorter than 60 min. Our results may be useful for helping to establish a new ECPR strategy for cardiac arrest patients. If we can predict the neurological outcome during ECPR, we might be able to develop innovative methods to further improve the neurological outcome of these patients.

The present study is associated with some limitations. Firstly, the present study is a single-centre, retrospective study with a small population. There was no ECPR protocol and the application of ECPR depended on the physician’s decision. Furthermore, it was not possible to evaluate the cerebral rSO_2_ value in all of the patients who underwent ECPR during this study period. The number of CPA cases was 420 during the study period. Second, only the patients whose cerebral rSO_2_ values were recorded during resuscitation were included in the present study. So, we could not evaluate the relationship between the cerebral rSO_2_ values and the cardiac index, the timing of ROSC and blood pressure. In this study, all patients did not get ROSC during the evaluation of the cerebral rSO_2_. Third, we did not evaluate the relationship between the cerebral rSO_2_ values and the blood sample parameters such as the SaO_2_, PaO_2_, PaCO_2_, haematocrit, and lactate values. Fourth, we could not evaluate brain function such as electroencephalogram. Moreover, we did not evaluate the cerebral rSO_2_ values in the pre-hospital setting. Recently, we developed a portable rSO_2_ monitor (HAND ai TOS®; TOSTEC Co.), which is 170 × 100 × 50 mm in size and 600 g in weight and which is small enough to carry in the pre-hospital settings. Thus, we can now measure the pre-hospital rSO_2_ values [8]. There is a need for a prospective multi-centre study that includes measurements from the pre-hospital setting.

## Conclusions

The cerebral rSO_2_ value during ECPR may predict the neurological outcome. The further evaluation of the validity of cerebral rSO_2_ monitoring during ECPR may lead to a new resuscitation strategy.
